# Identification of novel anti-tumor therapeutic target via proteomic characterization of ubiquitin receptor ADRM1/Rpn13

**DOI:** 10.1038/s41408-020-00398-9

**Published:** 2021-01-13

**Authors:** Yan Song, Ting Du, Arghya Ray, Krishan Chauhan, Mehmet Samur, Nikhil Munshi, Dharminder Chauhan, Kenneth C. Anderson

**Affiliations:** 1 LeBow Institute for Myeloma Therapeutics and Jerome Lipper Myeloma Center, Department of Medical Oncology, Dana-Farber Cancer Institute, Harvard Medical School, Boston, MA USA; 2 Department of Biomedical Engineering (BME), Wentworth Institute of Technology, Boston, MA USA

**Keywords:** Targeted therapies, Cell biology

Dear Editor,

Therapies targeting the 20S proteasome are the mainstay of treatment of patients with multiple myeloma (MM); however, resistance to proteasome inhibitor (PI) therapies commonly develops underlying relapse of disease^1–4^. PIs target the 20S proteasomal catalytic activities and, importantly, PI resistance has been linked to mutations in the β-5 chymotrypsin-like proteasome subunit in the 20S proteasome^5–7^, incomplete inhibition of 20S proteasomal activity^8^, and/or enhanced transcription and biogenesis of 20S proteasome subunits^9^. The 26S proteasome contains two major subcomplexes: the 20S proteolytic core particle and 19S regulatory particle. Although the 20S core particle harbors the protein degradative subunits, which are targeted by PIs, the 19S proteasome contains ubiquitin receptors (UbRs) and deubiquitinating enzymes that play a key role in initial binding, unfolding, and translocation of the protein substrates into the 20S proteasome complex for degradation^10^. One potential strategy to overcome PI resistance is to target 19S regulatory subunit rather than 20S core particle of the 26S proteasome complex. For example, 19S-associated UbR ADRM1/hRpn13 recognizes K48-linked polyubiquitinated proteins and facilitates their disassembly via deubiquitinating enzyme UCHL5, allowing for protein degradation via 20S proteasomal catalytic activities. We and others showed that blockade of hRpn13 triggers accumulation of polyubiquitinated proteins without affecting 20S proteasomal activities, inhibits MM cell growth, and overcomes PI resistance^11,12^. At present, hRpn13-modulated protein substrates and downstream signaling has not been delineated. Here we utilized multiplexed proteomics with tandem mass spectrometry, Gene Ontology (GO) enrichment, as well as pathway database Reactome to identify hRpn13-associated signaling molecules and delineate functionally significant biological pathways. The prognostic relevance of identified proteins was derived using Gene Expression Profiling (GEP) database on uniformly treated MM patients.

We performed CRISPR-Cas9 genome editing to generate stable *hRpn13*-knockout (hRpn13-KO) HCT116 cell lines. CRISPR-Cas9-mediated hRpn13 deletion was confirmed using PCR and sequencing, as well as immunoblot analysis (Supplementary Fig. 1A, B, respectively). As in our prior study^11^, a significant loss in cytotoxic activity of Rpn13 inhibitor RA190 was noted in hRpn13-KO vs. -wild type (WT) cells, further confirming deletion of hRpn13 in KO cells (Supplementary Fig. 1C). We next examined the proteomic alterations in the hRpn13-KO- vs. -WT cells. Expression patterns of proteins were analyzed using UniProt composite database and SEQUEST-based software platform, and a heat map was generated [>2-fold change in protein level was considered significant, confidence interval (CI) > 95%]. Proteins were quantified only from peptides with a summed SN threshold of ≥100 and isolation specificity of 0.5. The proteins were filtered to a <1% false discovery rate. Among 8766 proteins analyzed, 206 proteins were significantly downregulated (Fig. 1A), whereas 65 proteins were significantly upregulated (Fig. 1B), in hRpn13-KO cells compared to hRpn13-WT cells. Functionally relevant proteins were categorized using GO and Reactome pathway analysis (Supplementary Fig. 2). Targeted hRpn13 deletion upregulated multiple physiological pathways including those regulating amino acid biosynthesis and transportation, metabolism, proliferation, apoptosis, as well as tumor necrosis factor-α and fibroblast growth factor-induced signaling (e.g., KCNA3, ZNF236, SUMO2, IL1RAP, DPY30, AKT3, ANF484, BTF3, or CTSH). On the other hand, hRpn13 deletion decreased levels of proteins involved in cell adhesion, biological regulation, antigen binding, extracellular matrix interactions, and immunosuppressive signaling (e.g., TTC40, FCRLA, VGF, JARID2, S100A16, S100A14, ADIRF, hRpn13/ADRM1, IKZF1, and CYP2J2). Of note, hRpn13/ADRM1 plays a role in cell adhesion and extracellular matrix (ECM) interactions^13^, and importantly, many altered proteins in hRpn13/ADRM1-KO cells are related to adhesion/ECM interactions (e.g., ITGA2, ITGA3, ITGB1, ITGB4, LAMA3, LAMB2, LAMC2, ALCAM, CNTN1, GLG1, HLA-B, ITGB1, L1CAM, SELPLG, and VCAN).

**Fig. 1:**
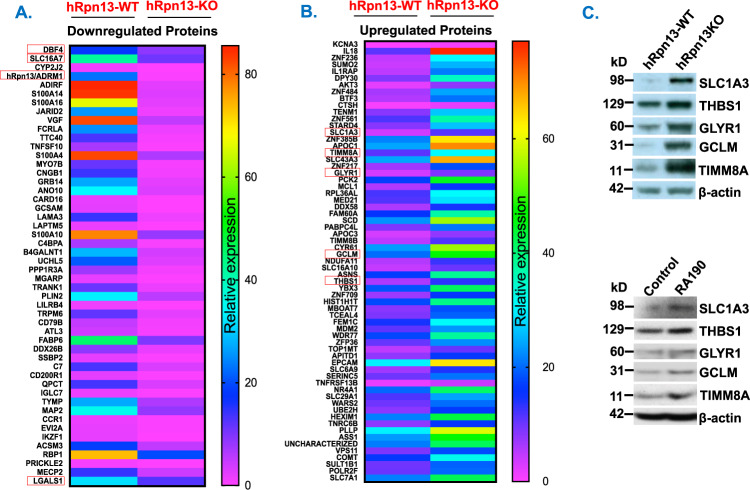
Analysis of hRpn13-mediated proteomic alterations. **(A**, **B)** CRISPR-Cas9-hRpn13-KO and -WT-HCT116 cells were subjected to proteomic analysis by multiplexed proteomics with tandem mass spectrometry. Expression patterns for 50 most downregulated or upregulated proteins were compared, and heat maps were generated (>2-fold change in protein level was considered significant, CI>95%). **(C)** Upper panel: Total protein lysates from hRpn13-WT and -KO cells were subjected to immunoblot analysis using antibodies against SLC1A3, THBS1, GLYR1, GCLM, TIMM8A, or β-actin. Lower panel: MM.1S cells were treated with DMSO control or hRpn13 inhibitor RA190 (0.5µM) for 16h; total protein extracts were subjected to immunoblot analysis using antibodies against SLC1A3, THBS1, GLYR1, GCLM, TIMM8A, or β-actin. Blots shown are representative of three independent experiments.

UbR hRpn13 mediates degradation of many cellular proteins via the 20S proteasome. Consistent with this notion, we hypothesized that proteins directly modulated by hRpn13 will fail to undergo degradation in the absence of hRpn13 and accumulate in cells. Using immunoblot analysis, we validated five such upregulated proteins (SLC1A3, THBS1, GLYR1, GCLM, and TIMM8A) in hRpn13-KO vs. -WT cells (Fig. 1C upper panel). Similar as hRpn13 deletion in HCT116 cells, treatment of MM.1S MM cells with hRpn13 inhibitor or their transfection with hRpn13-siRNA triggered an increase in SLC1A3, THBS1, GLYR1, GCLM, and TIMM8A levels (Fig. 1C lower panel and Supplementary Fig. 3A). Together, these data indicate that SLC1A3, THBS1, GLYR1, GCLM, and TIMM8A proteins are modulated by UbR hRpn13.

We next assessed the clinical relevance of hRpn13 deletion-triggered proteomic alterations in MM. As noted above, 271 proteins were significantly altered in hRpn13-KO vs. hRpn13-WT cells. We screened these 271 proteins against publicly available GEP datasets on MM patients (GSE6477, GSE13591, and GSE6691). We specifically analyzed the target molecule expression in MM patient samples vs. normal plasma cells, and whether expression levels correlate with overall survival in uniformly treated MM patients. Among 65 proteins upregulated in hRpn13-KO cells, 18 were expressed at significantly lower levels in MM patient samples vs. normal plasma cells and, importantly, 2 (SLC1A3 and THBS1) of these 18 proteins correlated with poor patient survival (Fig. 2A).

**Fig. 2:**
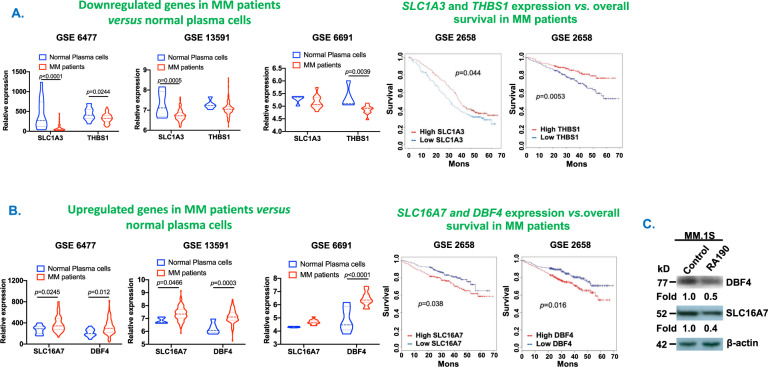
Clinical relevance of SLC1A3, THBS1, DBF4, and SLC16A7 in MM. **(A, box plot)** Gene expression data collected using Affymetrix Human Genome U133A [HG-U133A] Array platform based on normal plasma cells (*n*=15)s and newly diagnosed MM (*n*=69) patient samples (data accession number GSE6477). Similar analysis was obtained using gene expression data in GSE13591 and GSE6691. The data are presented as relative gene expression of indicated genes in MM patient vs. normal. The analysis of data was based on the information available on the following websites: http://www.canevolve.org/AnalysisResults/AnalysisResults.html. **(A, line graph)** Kaplan–Meier plots on the prognostic relevance of SLC1A3 and THBS1 gene expression on the overall survival of uniformly treated MM patients: patients with low SLC1A3 and THBS1 expression (blue line) had shorter survival, whereas patients with elevated SLC1A3 and THBS1 (red line) had longer survival. The gene expression analysis is based on Total Therapy-2 patients survival data collected using [HG-U133-Plus 2] Affymetrix Human Genome U133 Plus 2.0 Array platform (Data accession number GSE2658). **(B, box plot)** Gene expression data collected using Affymetrix Human Genome U133A [HG-U133A] Array platform based on normal plasma cells (*n*=15) and newly diagnosed MM patient samples (*n*=69) (data accession number GSE6477). Similar analysis was obtained using gene expression data on GSE13591 and GSE6691. Relative gene expression of indicated genes in MM patient samples vs. normal plasma cells is presented and data analysis was based on information from websites in ‘A’. **(B, line graph)** Kaplan–Meier plots on the prognostic relevance of SLC16A7 and DBF4 gene expression on overall survival of MM patients: patients with elevated SLC16A7 and DBF4 expression (red line) had shorter survival, whereas patients with low SLC16A7 and DBF4 expression (blue line) had longer survival. The gene expression analysis is based on Total Therapy-2 patients survival data collected using [HG-U133-Plus 2] Affymetrix Human Genome U133 Plus 2.0 Array platform (data accession number GSE2658). **(C)** MM.1S cells were treated with DMSO control or RA190 (0.5µM) for 16h; total protein extracts were subjected to immunoblot analysis using antibodies against DBF4, SLC16A7, or β-actin. Protein bands in immunoblot were quantified by densitometry using ImageJ and normalization with β-actin. Blots shown are representative of three independent experiments. Student’s *t*-test was utilized to derive statistical significance (Graph Pad PRISM version 6, La Jolla, CA, USA).

A recent study linked SLC1A3 to amino acid metabolism via modulation of aspartate metabolism by regulating glutamic and aspartic acid availability in tumor cells^14^. In our functional validation study using MM.1S MM cells, both biochemical (Fig. 1C lower panel) and genetic (small interfering RNA, siRNA) (Supplementary Fig. 3A) blockade of hRpn13 increases SLC1A3 levels. In addition, hRpn13 deletion also increased expression of other proteins (e.g., ASS1, ASNS, SLC7A1, and SLC16A10) involved in regulation of amino acid synthesis and transport (Supplementary Fig. 3B). Together, these findings suggest that targeting hRpn13 may modulate amino acid metabolism; however, further studies are needed to confirm a direct link between hRpn13 and amino acid metabolic pathways.

Similar to SLC1A3, we found that the adhesive glycoprotein Thrombospondin-1 (THBS1) is also expressed at low levels in MM patients and correlates with poor survival (Fig. 2A). Biochemical blockade of hRpn13 increases THBS1 levels in MM.1S (Fig. 1C lower panel). A previous study^15^ examined a correlation between treatment response and the levels of angiogenic factors including THBS1 in 96 patients with secretory MM: a significant increase in THBS1 concentrations was noted in the bone marrow plasma of patients achieving complete or very good partial response (reduction in monoclonal component by at least 90%), in contrast to those who showed partial or no response. These findings suggest that an increase in THBS1 levels may correlate with improved survival in MM. However, therapies increasing THBS1 may have limitations, as increased THBS1 levels have been implicated in the pathogenesis of MM bone disease^16,17^.

We next screened 206 of 271 proteins that were downregulated in Rpn13-KO cells in GEP database of MM patients (GSE6477, GSE13591, GSE6691). Results show that 32 of these 206 molecules were highly expressed in MM patient samples vs. normal plasma cells, and that 2 (SLC16A7 and DBF4) of 32 correlated with poor survival (Fig. 2B). SLC16A7 encodes for monocarboxylate transporter 2 (MCT2), a mediator of glycolysis contributing to elevated glycolytic metabolism in MM^18^. We found that biochemical inhibition of MCT2 induced cytotoxicity in MM.1S cells (Supplementary Fig. 3C). Similar as hRpn13 deletion, treatment of MM.1S cells with hRpn13 inhibitor RA190 or their transfection with hRpn13-siRNA decreased SLC16A7 levels (Fig. 2C, and data not shown).

Finally, hRpn13 deletion decreased DBF4, a regulatory subunit for CDC7 kinase. DBF4 regulates DNA replication and cell proliferation^19^, and our GEP database analysis shows that DBF4 is highly expressed in MM patient samples vs. normal plasma cells (Fig. 2B). Moreover, high DBF4 expression in MM patient samples is associated with poor survival (Fig. 2B). An earlier report showed that DBF4 inhibitor re-sensitizes melphalan-resistant cells to melphalan^20^. Importantly, we found that both biochemical and genetic blockade of hRpn13 decreased DBF4 expression in MM.1S cells (Fig. 2C, and data not shown). Together, these data suggest that: (1) SLC16A7 and DBF4 are downstream signaling targets of hRpn13; and (2) hRpn13-inhibition triggered MM cell death is associated with blockade of elevated glycolysis and DNA replication/cell growth *via* SLC16A7 and DBF4, respectively.

Collectively, our study utilized CRISPR gene-editing, biochemical, and molecular strategies to identify UbR hRpn13-mediated proteomic alterations. For proteomic studies, we generated CRISPR-Cas9 stable hRpn13-KO using HCT116 cells, as Rpn13 is essential for survival in MM cells and, importantly, we validated targets identified through proteomics studies in MM cells (Fig. 1C lower panel, Fig. 2C, and Supplementary Fig. 3A). Moreover, the potential prognostic and clinical significance was derived from multiple transcriptomic databases from MM patient samples and normal plasma cells, and the biologic significance validated using our in vitro MM models. We identified novel targets including SLC16A7, DBF4, SLC1A3, and/or THBS1, which may serve as prognostic biomarkers in MM. Although we found common downstream targets of hRpn13 in HCT116 vs. MM cells, it is likely that hRpn13 signaling/substrates may differ in cell-type context manner. Overall, our findings further support hRpn13-directed therapeutics, as well as preclinical evaluation of novel strategies targeting SLC16A, DBF4, SLC1A3, and THBS1, to enhance cytotoxicity and improve patient outcome in MM.

## Supplementary information

Supplemental figure
