# Supplementary material for: Identification of novel anti-tumor therapeutic target via proteomic characterization of ubiquitin receptor ADRM1/Rpn13
Source: Blood Cancer J. 2021 Jan 13;11(1):13. doi: 10.1038/s41408-020-00398-9 (PMC7806750; doi:10.1038/s41408-020-00398-9)
Supplement: Supplementary file 1 — Supplemental figure [file 41408_2020_398_MOESM1_ESM.pdf]

Supplementary Figure 3

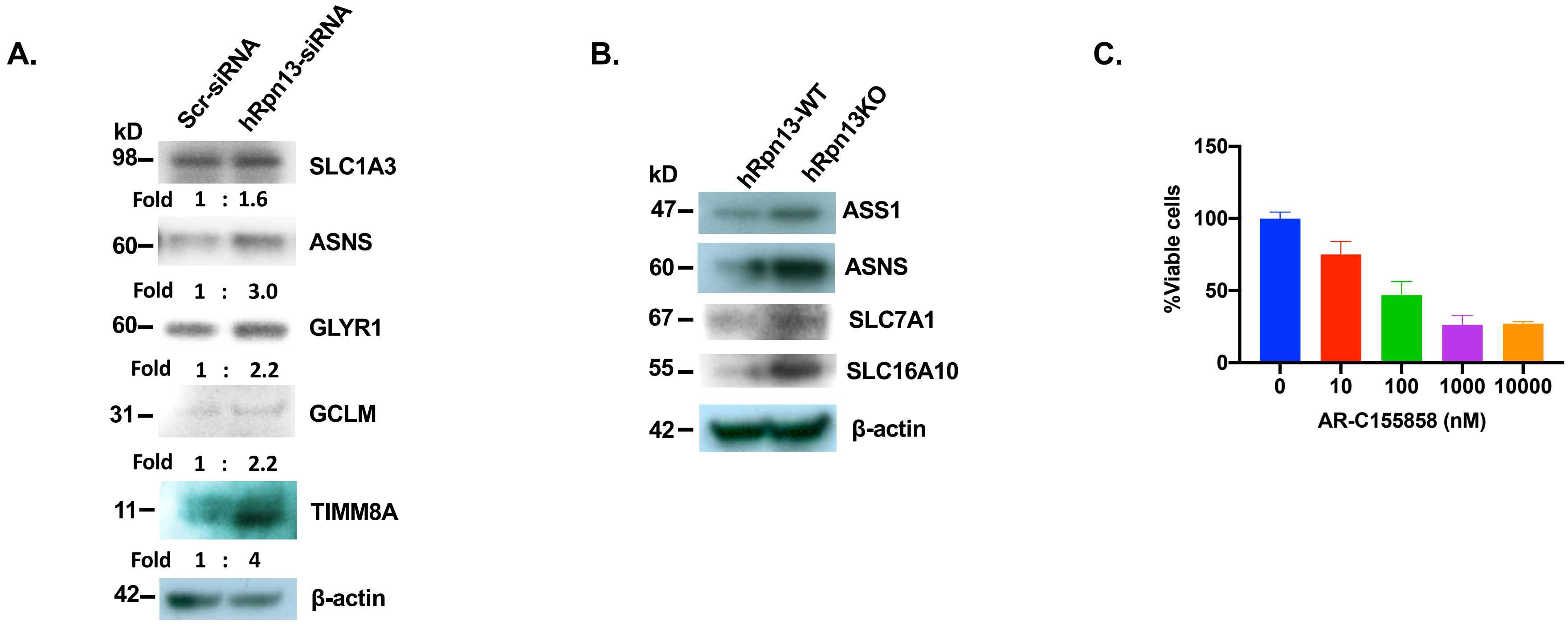

Supplementary Figure 3 **Validation of proteomic data (A)** MM.1S MM cells were transfected with either scr-siRNA or hRpn13-siRNA for 48h; total protein extracts were subjected to immunoblot analysis using antibodies against SLC1A3, ASNS, GLYR1, GCLM, TIMM8A, or β-actin. Protein bands in immunoblot were quantified by densitometry using ImageJ and normalization with β-actin. **(B)** Total protein lysates from hRpn13-WT and -KO HCT116 cells were subjected to immunoblot analysis using antibodies against ASS1, ASNS, SLC7A1, SLC16A10 or β-actin. **(C)** MM.1S MM cells were treated with various concentrations of MCT2 (SLC16A7) inhibitor AR-C155858 for 48h, followed by analysis of cell viability using WST assay (mean ± s.d.; $p < 0.0001$; n = 3).
